# Exploratory Pharmacokinetics of Geniposide in Rat Model of Cerebral Ischemia Orally Administered with or without Baicalin and/or Berberine

**DOI:** 10.1155/2013/349531

**Published:** 2013-12-03

**Authors:** Linmei Pan, Wenzhe Wang, Feiyan Shi, Jing Zhou, Meng Zhang, Huaxu Zhu, Mingfei Zeng

**Affiliations:** ^1^Separation Engineering of Chinese Traditional Medicine Compound, Nanjing University of Chinese Medicine, Nanjing 210028, China; ^2^Thermo Fisher Scientific, Shanghai, China

## Abstract

Huang-Lian-Jie-Du-Tang (HLJDT), a classical Chinese prescription, has been clinically employed to treat cerebral ischemia for thousands of years. Geniposide is the major active ingredient in HLJDT. The aim is to investigate the comparative evaluations on pharmacokinetics of geniposide in MCAO rats in pure geniposide, geniposide : berberine, and geniposide : berberine : baicalin. Obviously, the proportions of geniposide : berberine, geniposide : baicalin, and geniposide : berberine : baicalin were determined according to HLJDT. In our study, the cerebral ischemia model was reproduced by suture method in rats. The MCAO rats were randomly assigned to four therapy groups and orally administered with different prescription proportions of pure geniposide, geniposide : berberine, geniposide : baicalin, and geniposide : berberine : baicalin, respectively. The concentrations of geniposide in rat serum were determined using HPLC, and main pharmacokinetic parameters were investigated. The results indicated that the pharmacokinetics of geniposide in rat serum was nonlinear and there were significant differences between different groups. Berberine might hardly affect the absorption of geniposide, and baicalin could increase the absorption ability of geniposide. Meanwhile, berberine could decrease the absorption increase of baicalin on geniposide.

## 1. Introduction

**Figure 1:**
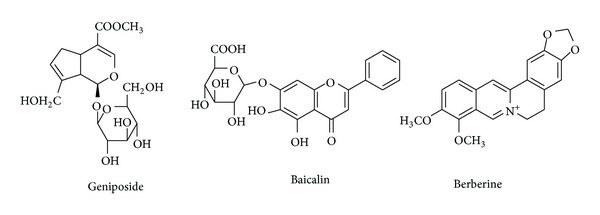
The structures of geniposide, baicalin, and berberine.

Huang-Lian-Jie-Du-Tang (HLJDT, or Oren-gedoku-to in Japanese), an important multiherb remedy in China and other Asian countries, has been used clinically to treat cerebral ischemia for decades [1–4]. It consists of four medicines including *Rhizome coptidis* (9 g), *Radix scutellariae* (6 g), *Cortex phellodendri* (6 g), and *Fructus gardenia* (9 g). Modern pharmacological studies have shown that HLJDT decoction can prevent cerebral ischemia and antioxidation [5, 6]. The Gardenia contains iridoid glycosides, in which geniposide has the highest content (content of which is up to 6%). Geniposide has a protective effect from loss of cerebral ischemia and reperfusion injury [7], but the mechanism is not clear. Geniposide inhibited focal cerebral ischemic injury and induced HIF-1*α* and HIF-1, which depends on apoptosis and expression of related gene RTP801mRNA, thereby reduced neuronal apoptosis. Geniposide has a strong antioxidative capacity and endothelial cell protective effect, which can reduce the endothelial cells of the vascular oxidation injury (Figure 1) [8]. In addition, geniposide had a certain analgesic and anti-inflammatory effect [9]. At present, researchers have studied the effects of some other ingredients on the pharmacokinetics and pharmacodynamics of geniposide [10–12].

There are two other major constitutes, alkaloids (berberine) and flavonoids (baicalin), existing in HLJDT (Figure 1). So it is necessary to investigate the pharmacokinetics of geniposide in different groups. The aim of this study is to explore whether the berberine and baicalin can affect the pharmacokinetic behavior of geniposide. It valued the formulation optimization design and clinical application of HLJDT. According to the proportion of pharmacodynamic ingredients of HLJDT, the study had comparatively evaluated the pharmacokinetics of geniposide in MCAO rats after oral administration of geniposide, geniposide : berberine, geniposide : baicalin, and geniposide : baicalin : berberine. The pharmacokinetics parameters were analyzed by software kinetica version 4.4 (Innaphase, MA, USA). The validated method is successfully applied to control the quality of geniposide and investigate interaction among other ingredients of HLJDT decoction in cerebrovascular disease.

## 2. Experiment

### 2.1. Materials and Reagents

Geniposide and paeoniflorin were purchased from National Institute for the Control of Pharmaceutical and Biological Products (Beijing, China). The extracts of geniposide, baicalin, and berberine were purchased from Zelang Biotechnology Company (Nanjing, China). Methanol used for HPLC was chromatographic grade (Han bang Company, Jiangsu, China). Deionized water was prepared in a Mill-Q academic water purification system (Millipore, Bedford, MA, USA). Glacial acetic acid was analytical grade, which was provided by Jiuyi Chemical Reagent Company (Shanghai, China). Heparin sodium was purchased from Sigma. Acetonitrile, HPLC grade was purchased from Merck.

### 2.2. Apparatus and Chromatographic Conditions

The High Performance Liquid Chromatography (HPLC) system consisted of a Waters 515 pump, DAD detector (Waters Association, Milford, MA, USA); N2000 LC chromatography workstation (Zhejiang University, China). The mobile phase was Acetonitrile (A) and 0.1% H_3_PO_4_ (B) with isocratic eluting (A : B = 12 : 88, v/v) at a flow rate of 1.0 mL/min. The detector operated at 238 nm. The injection volume was 20 *μ*L and the column temperature was 30°C.

### 2.3. Sample Treatment

The experiment mainly studied the influence of baicalin and berberine on the pharmacokinetic behavior of geniposide. According to the preliminary experiments, the average contents of geniposide, baicalin, and berberine were 22.82, 40.02, and 25.78 mg/g in HLJDT. Pure geniposide, geniposide : beberine, geniposide : baicalin, and geniposide : beberine : baicalin were weighed accurately on the basis of proportion in HLJDT, and then dissolved in 0.50% carboxymethyl cellulose sodium (CMC-Na) aqueous solution before use.

### 2.4. Animals

Male Sprague-Dawley (SD) rats were purchased from the slaccas experiment animal company (Shanghai, China). Animal welfare and experimental procedures were strictly in accordance with the Guide for the Care and Use of Laboratory Animals (US National Research Council, 1996) and the related ethics regulations of Nanjing University of Chinese Medicine.

Experiments were conducted in 290 ± 10 g SD rats under chloral hydrate anesthesia and fixed the rats on the operating table. A ventral midline incision was made in the neck. The omohyoid muscle was separated longitudinally and retracted laterally and isolated. A loose ligature was then placed around the external cartid artery. The external artery was permanently ligated rostrally. The vessel was ransected between the ligatures, and the remaining stump was reflected caudally. A piece of monofilament suture material was inserted into the lumen of the stump and advanced into the internal carotid artery. When completely advanced, the tip of the monofilament should block the blood flowing into the middle cerebral artery. The silk ligature was tied around the stump to secure the piece of monofilament. The skin irrcision was closed using wound clips. Four hours after surgery, as a successful replication of the model of MCAO, the body temperature will rise over 0.8°C, and rats showed visible symptoms of neurological deficit which are characterized by severe left-sided hemiparesis and right Horner's syndrome [13, 14]. It was mainly shown in reducing the activities, such as apathy, dumping to the right side when walking, keeping on rotating, and neurobiology score significantly increase. These were criteria for evaluating the ischemic insult. Rats, which did not show behavioral deficit, were excluded from the MCAO group, and the rest were divided into four subgroups randomly.

### 2.5. Biosample Collection

The MCAO rats were divided into four groups randomly, pure geniposide subgroup, geniposide : berberine subgroup, geniposide : baicalin subgroup, and geniposide : baicalin : berberine subgroup. Pure geniposide was dissolved in 0.5% CMC-Na aqueous solution and was oral to the pure geniposide subgroup rats (containing 142.28 mg geniposide/kg according to body weight). The geniposide : berberine subgroup, geniposide : baicalin subgroup, and geniposide : baicalin : berberine subgroup also received gavages of geniposide dissolved in 0.5% CMC-Na aqueous solution at the dosage of containing 142.28 mg geniposide/kg according to body weight. Blood samples (0.5 mL) were collected from the abdominal vein before dosing (to serve as a control) and at 0.083, 0.25, 0.5, 0.75, 1.0, 1.5, 2.0, 4.0, 6.0, 8.0, 12.0, and 24.0 h after drug administration, then immediately transferred into EP tube and centrifuged for 5 min at 5000 rpm in 4°C to separate serum. The serum was stored at −70°C after separation until assayed as described below.

Serum samples (200 *μ*L) were acidified with approximately 100 *μ*L of 0.01 mol/L acetic acid added to each of them. Then, each portion was vortexed with 800 *μ*L of methanol for 1 min and centrifuged at 5000 rpm for 10 min. The organic layer was transferred into an empty tube and was dried at 40°C under a nitrogen stream. The residue was dissolved in 100 *μ*L methanol. After centrifugation at 800 rpm for 10 min, fifty microliters supernatant was analyzed with HPLC.

### 2.6. Preparation of Standard Solutions and Quality Control Samples

The geniposide reference standards were accurately weighed and dissolved in methanol, and then diluted to appropriate concentration ranges for the establishment of calibration curves in rat serum. The concentration of reference solution was 9.26 *μ*g/mL. The paeoniflorin as internal standard was prepared as the same of geniposide and its concentration was 1.20 *μ*g/mL. These solutions were stored at 4°C. The geniposide reference standard solutions at seven different concentrations 0.15, 0.29, 0.58, 1.16, 2.32, 4.63, and 9.26 *μ*g/mL were prepared by spiking 100 *μ*L blank serum with appropriate volumes of the standard stock solution.

Recovery of the liquid-liquid extraction procedure was evaluated at low (0.15 *μ*g/mL), medium (0.58 *μ*g/mL), and high (2.32 *μ*g/mL) concentrations for geniposide. It was calculated by comparing the mean peak area (*n* = 5 at each concentration) of the extracted quality control sample with that of the unextracted standard solution containing the equivalent amount of analyses.

Quality control (QC) samples were used for the study of intraday and interday accuracy and precision; extraction recovery and stability were prepared in the same way as above and were prepared from blank serum at concentrations of 0.15, 0.58, and 2.32 *μ*g/mL; five replicates were analyzed in each of the three analytical runs. The accuracy was expressed by the relative error (RE) and the precision was evaluated by the relative standard deviation (RSD).

The stability of geniposide was evaluated under mimicking conditions likely to be encountered during sample storage and the analytical process by analyzing five replicates of QC samples for the analysis. The freeze-thaw stability was determined after one freeze and thaw cycle. The QC samples were stored at −20°C for 24 h and thawed unassisted at room temperature.

### 2.7. Data Analysis

The different serum concentrations of geniposide were expressed as means ± standard deviation (SD) and the mean concentration-time curve was plotted. All data were analyzed by software kinetica version 4.4 (Innaphase, MA, USA) to obtain the relative pharmacokinetic parameters. Statistical analysis was performed using the SPSS Version 13.0 (SPSS Inc., Chicago, IL). Differences in numerical variables among groups were analyzed with one-way analysis of variance (ANOVA) LSD-t and SNK-q. A *P* value of less than 0.05 was considered statistically significant.

## 3. Results

### 3.1. Selectivity

**Figure 2:**
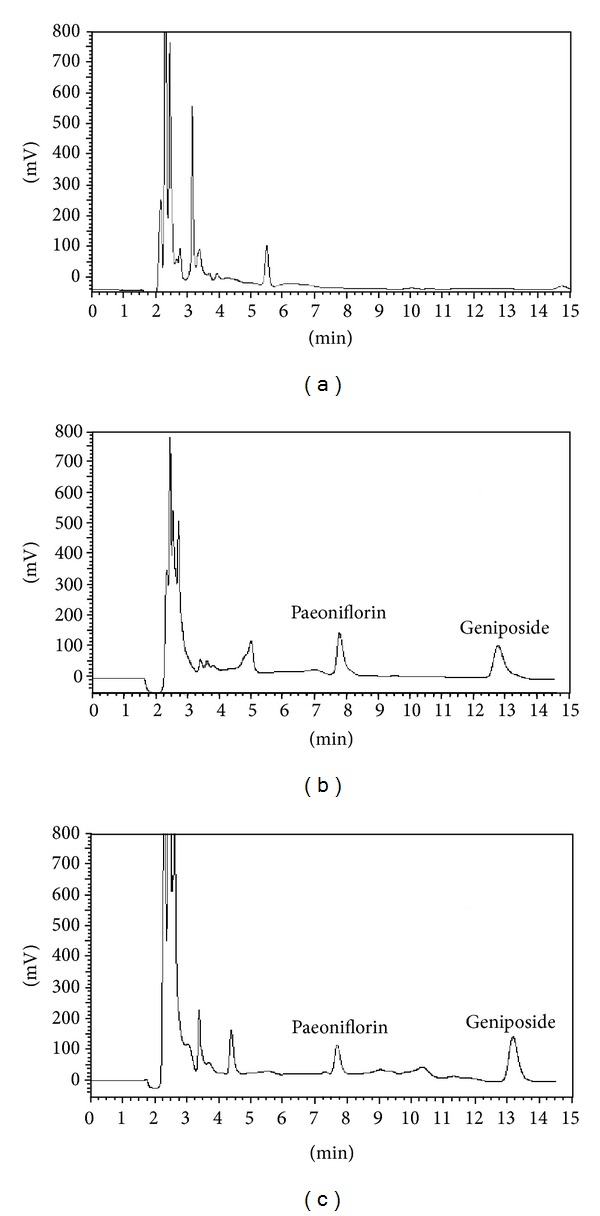
Typical chromatograms for the determination of geniposides: (a) chromatogram of a blank serum sample, (b) chromatograms of a serum sample spiked with paeoniflorin and geniposide, and (c) chromatogram of the serum sample of an MCAO rat taken 1 h after the oral administration of the geniposide.

The selectivity of the method was evaluated by analyzing blank serum samples prior to administration. The chromatograms were free of interfering peak at the retention time of geniposide (13.3988 min); the retention time paeoniflorin (8.392 min) of Figure 2 showed the representative chromatograms of blank serum sample (a), serum sample spiked with geniposide and serum sample spiked with paeoniflorin (b), and serum samples 1 h after oral administration of geniposide (c).

### 3.2. Linearity and Lower Limit of Quantification

Each calibration curve was constructed with six different concentrations by plotting the peak areas ratios of geniposide versus the concentration of geniposide using linear regression. Good linearity was obtained from 0.15 to 9.26 *μ*g/mL. The lower limit of quantification (LLOQ) for geniposide was 0.15 *μ*g/mL.

### 3.3. Accuracy and Precision

The intra- and interday precision and accuracy were determined by replicate analyses of QC samples continuously on the same day (intraday) for 5 days (inter-day), respectively. The intra-and interday precision and accuracy were shown in Table 1. The precision of geniposide calculated as the relative standard deviation (RSD) at various concentrations was lower than 15% for intra- and interday assays, and the accuracy was within 15% for QC samples. The results demonstrated that the precision and accuracy of this method were acceptable.

### 3.4. Recovery

The mean (±SD) recovery for geniposide from rat serum was 85.34 ± 2.04%, 81.92 ± 2.23%, 78.75 ± 3.69% at 2.32, 0.58, 0.15 *μ*g/mL, respectively., 0.15, 0.58, and 2.32.

### 3.5. Stability

The stability of geniposide was determined by analyzing QC samples at three concentrations exposed to encounter during sample storage. QC samples were frozen and stored at −20°C for a week. Freeze-thaw stability was determined after one cycle of freezing and thawing, the corresponding relative errors were less than ±10% for samples at the concentrations of 4.36, 3.45, and 8.79 *μ*g/mL, respectively. Geniposide showed good stability in one week after storage at −20°C for 7 days, the concentration of geniposide in serum would deviate to less than ±15% from those in freshly spiked serum.

### 3.6. Pharmacokinetics Study

**Figure 3:**
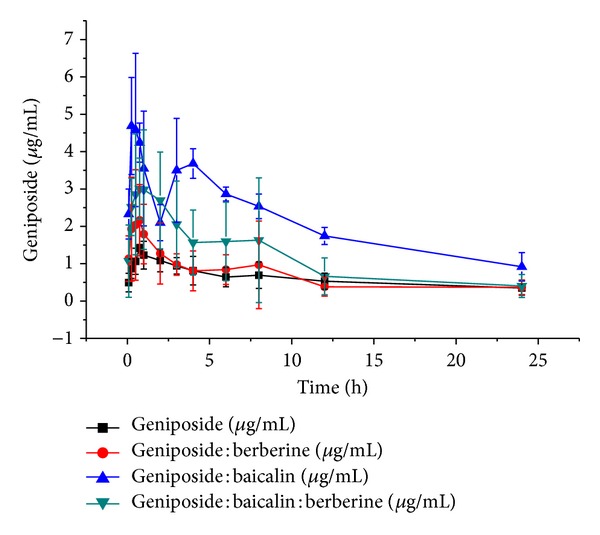
The serum concentration-time curve of geniposide in rats after oral administration of pure geniposide, geniposide : berberine, geniposide : baicalin, and geniposide : baicalin : berberine to MCAO rats.

HLJDT contains effective parts of three categories: alkaloids, flavonoids, and iridoid glycosides, three active ingredients: berberine, baicalin, and geniposide, and so forth [15]. The developed and validated HPLC method was used to determine geniposide in rat serum after oral administration of pure geniposide, geniposide : berberine, geniposide : baicalin, and geniposide : baicalin : berberine. The mean serum concentration-time curve profiles were illustrated in Figure 3 and its pharmacokinetics data were shown in Table 2. The concentration-time profile demonstrated bimodal phenomenon; the first peak occurred at about 0.75 h and the second at 8 h after oral administration of pure geniposide. Compared with pure geniposide, bimodal phenomenon of geniposide : berberine, geniposide : baicalin, and geniposide : baicalin : berberine in MCAO rats serum also existed, but two peak times were advanced, and the values of AUC_(0−*t*)_, AUC_(0−*∞*)_, and AUMC_(0−*t*)_, *C*_max⁡_ were significantly increased after oral administration of geniposide : baicalin (*P* < 0.01). These results indicated that baicalin increased the absorption of geniposide. Pharmacokinetic comparison of geniposide on MCAO rats after administration of geniposide and geniposide : berberine illustrated that the existence of berberine might hardly affect the pharmacokinetic behavior of geniposide.

In order to observe the pharmacokinetic behavior of geniposide in combination with baicalin and berberine, the pharmacokinetics of geniposide with pure geniposide, geniposide : baicalin, and geniposide : baicalin : berberine in MCAO rats were compared. The serum concentrations of geniposide were determined and pharmacokinetic parameters were estimated (Table 2). As shown in Figure 3 and Table 2, the serum profile demonstrated that the absorption ability of geniposide in geniposide : baicalin : berberine group was higher than pure geniposide group and lower than geniposide : baicalin group. The lower concentration and decreased AUC_(0−*∞*)_ of geniposide were found in MCAO rats given geniposide : baicalin : berberine compared to geniposide : baicalin. These results suggested that berberine decreased the absorption of geniposide : baicalin.

## 4. Discussion

Huang-Lian-Jie-Du-Tang, the representative medicine for heat-clearing and detoxicating, has been used to treat cerebral ischemia for thousands of years in China. In the past few years, geniposide was confirmed as the main pharmacodynamic ingredient anticerebral ischemia in HLJDT [16]. After comparing of an organic solvent such as acetonitrile, ethyl acetate, chloroform, methanol [17–22] and through comprehensive comparison recovery, impurity interference and other factors, it is ultimately found that methanol can basically comply with the requirements of the experiment, so the selection of the organic solvent of methanol precipitates protein. Geniposide in rat serum determination investigated geniposide content in the serum level of the HPLC method; the method provides highly sensitive, specific, reliable data. The requirements were applicable to the determination of biological samples researching HLJDT pharmacokinetics, providing a reliable method.

It investigated pharmacokinetic differences of coadministration geniposide with baicalin and/or berberine changing by time in cerebral ischemia rats. Using kinetica version 4.4 and SPSS Version 13.0 to process serum concentration data and using the method of statistical moments we calculated pharmacokinetic parameters [23–25]. As shown in Figure 3 and Table 2, the concentration-time profile of geniposide : berberine, geniposide : baicalin, and geniposide : baicalin : berberine in MCAO rats serum demonstrated bimodal phenomenon. The bimodal phenomenon might be resulted from some circulations such as hepatoenteral circulation which was consistent with other published conclusions [26, 27]. The peak times of geniposide : baicalin and geniposide : baiclin : berberine were advanced, and the advanced times of geniposide : baicalin were longer than that of geniposide : baiclin : berberine. The values of AUC_(0 − *t*)_, AUC_(0 − *∞*)_, and *C*_max⁡_ of geniposide : berberine were 16.04 ± 4.61, 21.32 ± 9.36, and 2.99 ± 0.87, which were similar to those of pure geniposide (14.59 ± 4.24, 23.63 ± 13.02, and 1.48 ± 0.19) and lower than that of geniposide : baicalin : berberine (26.88 ± 13.83, 30.61 ± 14.56, and 3.99 ± 1.08) and geniposide : baicalin (49.59 ± 2.68, 59.00 ± 10.59, and 5.68 ± 1.19). The data demonstrated that coadministration of geniposide with baicalin, the bioavailability of geniposide, increased significantly in MCAO rats body. It might be because baicalin inhibited the hydrolysis of geniposide. Berberine showed no significant influence on behaviors in the pharmacokinetics of geniposide in MCAO rats, but it decreased the bioavailability of coadministration of geniposide with baicalin. It might be because berberine partly neutralized baicalin and the hydrolysis on geniposide was enhanced.

At the same time, we studied the pharmacokinetics of geniposide with or without baicalin and/or berberine in normal rats. The results had some differences from that of the MCAO rats. Compared with normal rats, in MCAO rat, the time to peak (*t*_max⁡_) was shorter, apparent volume of distribution (*V*) and concentration to peak (*C*_max⁡_) were higher, the dwell time (MRT) was longer, clearance rate (CL) was lower, and so on, indicating that the absorption effects of geniposide in MCAO rats were better than that of normal rats. Hou et al. [28] considered that the geniposide could be hydrolyzed by sulfatase, and *β*-glucuronidase held that the hydrolyzed enzymes might be reduced in MCAO rats. However, in the future, more experiments such as tissue distribution and metabolic pathway of geniposide in the body should be taken out for the purpose of exploring its absorption and metabolism mechanism.

## 5. Conclusions

The animal study showed significant comparative pharmacokinetics of geniposide alone or in combination with baicalin and/or berberine in rat model of cerebral ischemia. Coadministration of geniposide with baicalin could significantly increase the AUC_(0−*t*)_, AUC_(0−*∞*)_, AUMC_(0−*t*)_, and *C*_max⁡_. In the meantime, coadministration of geniposide with berberine could not significantly affect the pharmacokinetic parameters. However, the pharmacokinetic parameters of geniposide : baicalin were significantly decreased by berberine.

## Figures and Tables

**Table 1:** Precision and accuracy of the determination of geniposide in rat serum (interday *n* = 5; intraday *n* = 5).

Concentration (*μ*g/mL)	Intraday	Interday
	RSD (%)	RSD (%)
2.32	1.34	6.68
0.58	2.74	5.06
0.15	3.37	6.31

**Table 2:** Pharmacokinetic differences of oral administration of geniposide, geniposide : baicalin, geniposide : berberine, and geniposide : baicalin : berberine to MCAO rats.

Parameters	Geniposide	Geniposide : baicalin	Geniposide : berberine	Geniposide : baicalin : berberine
AUC_(0–*t*)_ (mg/L∗h)	14.59 ± 4.24	49.59 ± 2.68**	16.04 ± 4.61	26.88 ± 13.83
AUC_(0–*∞*)_ (mg/L∗h)	23.63 ± 13.02	59.00 ± 10.59**	21.32 ± 9.36	30.61 ± 14.56
AUMC_(0–*t*)_	136.73 ± 54.16	435.39 ± 48.12**	135.39 ± 39.93	203.81 ± 116.58
AUMC_(0–*∞*)_	235.03 ± 196.14	589.97 ± 73.48	188.91 ± 55.26	297.80 ± 137.63
MRT_(0–*t*)_ (h)	9.15 ± 1.38	8.77 ± 0.72	8.47 ± 1.24	7.42 ± 0.97*
MRT_(0–*∞*)_ (h)	22.52 ± 17.38	13.13 ± 4.83	15.81 ± 6.91	11.76 ± 4.95
VRT_(0–*t*)_ (h^2^)	49.40 ± 7.29	43.48 ± 10.59	53.26 ± 20.10	41.71 ± 15.44
VRT_(0–*∞*)_ (h^2^)	770.40 ± 1189.48	177.17 ± 164.06	316.07 ± 248.52	188.61 ± 178.69
*t*_1/2*z*_ (h)	15.11 ± 12.73	8.47 ± 3.45	10.41 ± 6.17	8.42 ± 4.45
*T*_max⁡_ ( h)	0.96 ± 0.53	0.55 ± 0.19	0.88 ± 0.65	0.60 ± 0.39
CL*z*/*F* (L/h/kg)	8.05 ± 4.76	2.47 ± 0.37	7.55 ± 2.52	5.38 ± 2.05
V*z*/*F* (L/kg)	119.31 ± 41.06	28.72 ± 6.09**	97.81 ± 49.63	66.23 ± 43.19
*C*_max⁡_ (mg/L)	1.48 ± 0.19	5.68 ± 1.19**	2.99 ± 0.87**	3.99 ± 1.08**

Values are given as means ± SD of 6 rats.

*P < 0.05, compared with pure geniposide group.

**P < 0.01, compared with pure geniposide group.
